# Development of a Novel Q-body Using an In Vivo Site-Specific Unnatural Amino Acid Incorporation System

**DOI:** 10.3390/s18082519

**Published:** 2018-08-01

**Authors:** Yoichi Kurumida, Nobuhiro Hayashi

**Affiliations:** Department of Life Science, School and Graduate School of Bioscience and Biotechnology, Tokyo Institute of Technology, Meguro-ku, Tokyo 152-8550, Japan; ykurumida@bio.titech.ac.jp

**Keywords:** Q-body, quenchbody, fluorescent biosensor, in vivo site-specifically unnatural amino acid incorporated system, HIV Nef

## Abstract

A Q-body capable of detecting target molecules in solutions could serve as a simple molecular detection tool. The position of the fluorescent dye in a Q-body affects sensitivity and therefore must be optimized. This report describes the development of Nef Q-bodies that recognize Nef protein, one of the human immunodeficiency virus (HIV)’s gene products, in which fluorescent dye molecules were placed at various positions using an in vivo unnatural amino acid incorporation system. A maximum change in fluorescence intensity of 2-fold was observed after optimization of the dye position. During the process, some tryptophan residues of the antibody were found to quench the fluorescence. Moreover, analysis of the epitope indicated that some amino acid residues of the antigen located near the epitope affected the fluorescence intensity.

## 1. Introduction

A Q-body is a recombinant antibody incorporating a fluorescent dye that can be used as a biosensor to detect proteins or small molecules based on an antigen-dependent change in quenching [1,2]. The most common methods employing antibodies to detect antigens are the enzyme-linked immunosorbent assay (ELISA) and western blotting (WB). However, both methods require time-consuming incubation, multistep treatments and antigen immobilization procedures. In addition, antibodies recognizing tertiary structures of target antigens sometimes cannot be used in these methods [3]. By contrast, assays using Q-bodies require only a fluorescence measurement after the antigen and Q-body are mixed in solution as a single step process. This would offer great advantages available for Kd measurements, some in-line readout in a process, etc. As such, the Q-body is considered a new type of immune-biosensor [1].

Some tryptophan (Trp) residues of antibodies are known to quench the fluorescence of dye molecules incorporated into antibodies [1,4,5]. When the antigen binds to the Q-body, the dye is no longer quenched because of increased separation in distance between the dye and some moiety causing the quench, and the fluorescent signal intensity increases. According to some past studies [6,7], changes in fluorescent response depends on the incorporation position and type of fluorescence dye.

Generally, a protein is artificially modified using an NHS-ester reaction via a primary amino group or thiol-maleimide coupling via a cysteine residue [8,9]. Site-specific modification is important for obtaining high-level responses because the position of the fluorescent dye molecule affects the response rate [6]. However, site-specific modification using the abovementioned methods is difficult in cases of antibodies harboring their own primary amino groups or cysteine residues. Some researchers addressed this problem by incorporating a fluorescent dye at a specific site of a Q-body using a cell-free transcription-translation system [1]. Although this is a convenient tool, high cost has prevented its widespread use as a general method.

To develop a high-performance Q-body, some researchers have examined various fluorescent dyes and incorporation positions [6]. Q-bodies are typically synthesized using a cell-free transcription-translation system [1], but new methods for incorporating fluorescence dye molecules in antibodies at nonspecific sites using cysteine residues [7] or formation of complexes of labeled protein and non-labeled antibody [10] have also been developed. In terms of applications, Q-bodies are useful not only as biosensors in solution but also as low-background immunostaining tools [11].

In this study, the unnatural amino acid (UAA) 3-azido-l-tyrosine was incorporated into antibodies using an in vivo site-specific UAA incorporation system [12,13,14]. The original system had been developed as an in vitro site-specific incorporation system, and had its own advantages. The incorporation site was strictly restricted, and number of the incorporation position was also exactly controlled as a site. These features were available to ensure that a molecule was stoichiometrically harboring a label, and to avoid some blockage of the original function. Despite the big advantage, because of high skills required for the usage, the technique was hard for beginners to use easily. In such situations, the in vivo system based on the original in vitro system had been developed, and was easy for beginners to use. The resulting antibodies were labeled using DBCO-Cy3 to develop the Q-bodies. In this study, an antibody that recognizes the human immunodeficiency virus (HIV) antigen Nef was used as a prototype.

## 2. Materials and Methods

### 2.1. Materials

**Figure 1:**
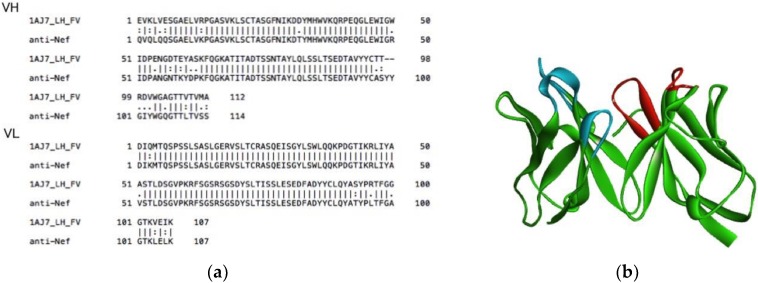
The structure of the anti-Nef antibody as predicted using homology modeling. (**a**) Alignment of the anti-Nef antibody and template (PDB code 1AJ7). (**b**) Model of the anti-Nef antibody. Green: framework, Blue: complementarity-determining region (CDR) of VH, Red: CDR of VL.

KOD-FX-neo DNA polymerase was purchased from Toyobo (Osaka, Japan). The Wizard^®^ Plus SV Miniprep DNA purification system was purchased from Promega Corporation (Madison, WI, USA). Bovine serum albumin (BSA) and DBCO-Cy3 were purchased from Sigma (St. Louis, MO, USA). Lysozyme was purchased from Wako (Osaka, Japan). Oligonucleotides were purchased from Eurofins Genomics (Tokyo, Japan). JM109 competent cells, MV1184 competent cells, and T4 DNA ligase were purchased from Takara (Osaka, Japan). Restriction enzymes and BL21(DE3) competent cells were purchased from New England Biolabs (Ipswich, MA, USA). 3-Azido-l-tyrosine was purchased from Watanabe Chemical Industries (Hiroshima, Japan). IgG Sepharose 6 Fast Flow, HiTrap Desalting and HisTrap HP were purchased from GE Healthcare Japan (Tokyo, Japan). Advanced Protein Assay was purchased from Cytoskeleton Inc (Denver, CO, USA). Anti-streptavidin IgG-HRP was purchased from Fitzgerald (Acton, MA, USA). Anti-His-probe(H-3) IgG-HRP (sc-8036) and mouse anti-rabbit IgG-HRP (sc-2357) were purchased from Santa Cruz Biotechnology (Dallas, TX, USA). Immobilon Western HRP substrate was purchased from Merck Millipore (Tokyo, Japan). A single-chain variable fragment (scFv) for which the antigen is HIV Nef protein was isolated in vitro from a constructed mouse antibody library [15], and the amino acid sequence is shown in Figure 1a. The gene encoding the Nef protein (HIV-1 NL43) was kindly provided by Y. Fujii [16].

### 2.2. Homology Modeling

The amino acid sequence of the anti-Nef antibody was analyzed according to the international ImMunoGeneTics scheme [17], and the tertiary structure was predicted in reference to Protein Data Bank (PDB) [18]:1AJ7 [19] as a template for the overall molecule, variable domain of the heavy chain (VH), and variable domain of the light chain (VL) (sequence similarity 96.7%, 94.5%, and 98.9%, respectively) (Figure 1a). The sequence analyses, homology modeling, and figure generation were carried out using Discovery Studio (Accelrys.co.jp).

### 2.3. Construction of Mutant Antibody and Nef Genes

Point-mutation expression vectors except for Nef Δ2-11 were constructed using the site-directed mutagenesis method [20] with the primers and templates summarized in Table 1 and Table 2.

The polymerase chain reaction amplification products were treated with the restriction enzyme DpnI. The deletion mutant Nef Δ2-11 expression vector was constructed via PCR with the primers and templates summarized in Table 1 and Table 2. The PCR-amplification product and pET23d were treated with the restriction enzymes NcoI and NotI and ligated using T4 DNA ligase. The above products were then transformed into JM109 competent cells. Each of the resulting plasmids was prepared using the Wizard^®^ Plus SV Miniprep DNA purification system. Confirmation of the DNA sequences were performed by general DNA sequencers (Genetic Analyzer, Thermo Fisher Scientific) at biomaterials analysis division at Tokyo Institute of Technology.

### 2.4. Antibody Expression and Purification 

MV1184 co-transformed with pRP_WB-Sup&R3YS [13] was transformed with the antibody expression vectors described above, and the transformants were grown at 30 °C in 2× YT broth containing ampicillin (100 µg/mL) and chloramphenicol (34 µg/mL) for 20 h as a pre-culture. The pre-culture was then grown at 30 °C in 2× YT broth containing ampicillin (100 µg/mL), chloramphenicol (34 µg/mL), 3-azido-l-tyrosine (200 µg/mL), and IPTG (1 mM) for 20 h in the dark. The precipitates were collected by centrifugation at 6500× *g* for 10 min at 4 °C and resuspended in phosphate-buffered saline (PBS) containing lysozyme (1 mg/mL) and DNase (1 µg/mL), then lysed by sonication. The lysate supernatant was used for purification of antibodies using an IgG-conjugated column. Antibodies bound to the column were eluted with citric acid-NaOH (20 mM; pH 2.5). After substitution of the buffer and concentration of the eluted fraction, DBCO-Cy3 (200 µM) was added to the purified antibodies in dimethyl sulfoxide (50% solution), and the mixture was incubated overnight at 4 °C. To remove the unbound dye, the reaction buffer was exchanged with PBS (pH 7.4) using a desalting column. The labeled antibodies were resolved by SDS-PAGE and detected by fluorescence imaging of the gel using an image analyzer (Typhoon FLA 9500, GE, Tokyo, Japan). The concentration of the antibodies was measured using Advanced Protein Assay. The concentration of the labeled antibodies was measured using an Infinite^®^ 200 PRO (Tecan, Japan) at an excitation wavelength of 490 nm and emission wavelength of 560 to 580 nm.

### 2.5. Antigen Expression and Purification 

*Escherichia coli* BL21(DE3) cells were transformed with respective plasmids for the expression of Nef wt, Nef Δ2-11, and Nef W13A. Bacteria were grown at 37 °C in Luria–Bertani (LB) medium containing ampicillin (100 µg/mL). When the culture reached mid-logarithmic phase, IPTG (1 mM) was added, and the culture was continued for 3 additional hours. The precipitates were collected by centrifugation at 6500× *g* for 10 min at 4 °C, resuspended in Tris-HCl buffer (pH 8.0) containing NaCl (500 mM), lysozyme (1 mg/mL), and DNase (1 µg/mL), and then lysed by sonication. The lysate supernatant was used for purification using a 1-mL HISTrap column. Protein bound to the column was eluted with imidazole (200 mM).

### 2.6. Fluorescence Measurements

Cy3-labeled antibodies (5 nM) in PBS containing 0.1% Tween 20 (PBSt) containing BSA (1%) and Nef (0–3.2 µM) were dispensed into a Nunc MicroWell™ 96-well microplate (Thermo Scientific, Tokyo, Japan). The solution was incubated for 2 min at room temperature before spectral measurement with reference to a previous study [5]. Fluorescence intensity was measured using an Infinite^®^ 200 PRO (Tecan) at an excitation wavelength of 490 nm, and at emission wavelength from 560 nm to 580 nm. The excitation and emission slit widths were set to 1.0 nm. The fluorescence intensities, which averaged 566 to 575 nm for each sample, were plotted. Response curves were fitted using Image J software (National Institutes of Health, Bethesda, MD, USA), and data were calculated from the curve fitting to a 4-parameter logistic equation:

(1) $$y=a+\left(d-a\right)/(1+\left(x/c{)}^{b}\right)$$

### 2.7. Western Blotting

Nef wt and Nef Δ2-11 were resolved by SDS-PAGE [21], and the resulting bands were transferred to a polyvinylidene difluoride membrane for Western blotting. The membrane was blocked with skim milk (5%) in Tris-buffered saline containing 0.1% Tween 20 at room temperature for 1 h. Anti-Nef and anti-His-tag antibodies were used for detection of Nef protein and His-tag, respectively. Anti-streptavidin IgG-HRP was used for detection of anti-Nef antibody. Western blotting signals were detected by chemiluminescence.

### 2.8. ELISA

Maxisorp microtiter plates (Nunc, Roskilde, Denmark) were coated with 50 μL of Nef protein (20 mg/L) in PBS and incubated overnight at 4 °C. The plates were washed twice with PBSt and blocked with 200 μL of BSA (2%) in PBS for 3 h at 37 °C. The plates were then washed twice with PBS. Anti-Nef antibody in PBSt was added, and the plates were incubated for 1 h at room temperature then washed twice with PBSt. Bound anti-Nef antibodies were detected with a 1:2000 dilution of mouse anti-rabbit IgG-HRP. The plates were washed four times with PBSt, and tetramethylbenzidine (TMB) substrate solution was added, followed by addition of stopping solution (1 M HCl) after 20 min of incubation at room temperature. The absorbance at 405 nm was monitored using an iMark microplate reader (Bio-Rad, Tokyo, Japan).

## 3. Results

### 3.1. Homology Modeling of anti-Nef Antibody

The tertiary structure of the anti-Nef antibody was predicted by homology modeling [22,23] using Discovery Studio software (Figure 1b). As a result of the homology search, an anti-Nef antibody model with the antibody (PDB code 1AJ7) was obtained as a template, and the root mean square deviation of the backbones was 0.49 nm. Analysis of a Ramachandran plot [24] for the structure revealed that 215 residues were in the most favored region, and four Gly residues were in a generously allowed region.

### 3.2. Construction of the Nef Q-body

**Figure 2:**
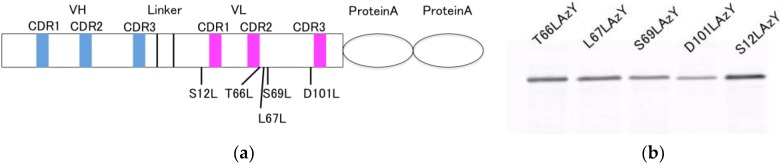
Expression of Cy3-labeled anti-Nef antibody constructed using the in vivo UAA incorporation system. (**a**) Scheme of the anti-Nef antibody gene. (**b**) Fluorescence image of SDS-PAGE of the expressed and modified Nef Q-bodies.

The Nef Q-body gene was constructed by site-directed mutagenesis of the template gene. Some mutagenesis positions were within four residues of the complementarity-determining regions (CDRs) (T66L, L67L, S69L, S101L), and one was on the opposite side from the CDRs (S12L) (Figure 2a). Five mutants (T66LAzY, L67LAzY, S69LAzY, S101LAzY, and S12LAzY) were constructed by changing the codon encoding the respective amino acid residue to the amber codon.

Antibodies site-specifically harboring 3-azido-l-tyrosine incorporated using the in vivo introduction system with the amber codon were produced (Table 1). After induction of expression in host cells, the antibodies were purified by the interior protein A binding to the IgG-conjugated column. The purified antibodies were modified by DBCO-Cy3 via the 3-azido-l-tyrosine residue. The Q-bodies were resolved by SDS-PAGE and detected by fluorescence imaging of the gel (Figure 2b). The labeling efficiencies of the UAA incorporations were roughly estimated to be about 20–50% by protein quantification assay. Difference of the efficiencies of each sample are thought to be resulted from the tertiary structural surrounding environment of each incorporated site. However, because the exact elucidation is difficult and is the other issue, experimental data of this time have been shown with consideration of the effect although non-labeled molecules might compete with the labeled ones. 

### 3.3. Antigen-dependent Changes in Fluorescence Intensity of the Labeled Antibodies

**Figure 3:**
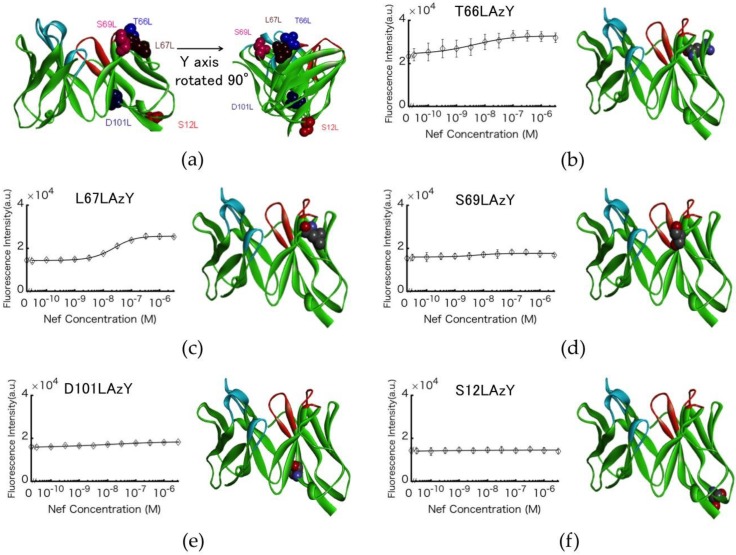
Antigen-dependent enhancement of Nef Q-body fluorescence. (**a**) Position of the mutated residues of the anti-Nef antibody. (**b**–**f**) Schematic illustration of the structure of the Nef Q-body (right). Titration curves of the normalized fluorescence intensity of the Nef Q-body (left). Error bars represent ±1 S.D. (n = 3).

Five types of labeled antibody were mixed with 0–3.2 µM Nef wt. Four of the clones (T66LAzY, L67LAzY, S69LAzY, and D101LAzY) exhibited an antigen-dependent change in fluorescence intensity (Figure 3a–f). The fluorescence intensity of S12LAzY did not change. The Q-body exhibiting the largest change was L67LAzY (1.8-fold).

### 3.4. Involvement of Internal Trp Residues in the Quenching of Cy3 at Position L67L

**Figure 4:**
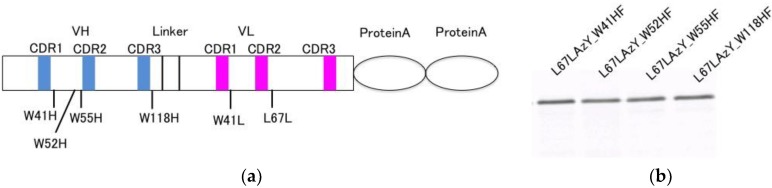
Expression of the Cy3-labeled mutant of L67LAzY constructed using the in vivo UAA incorporation system. (**a**) Scheme of the anti-Nef antibody gene. (**b**) Fluorescence image of SDS-PAGE of the expressed and modified Nef Q-bodies.

Trp-to-Phe mutants were constructed to elucidate the involvement of Trp residues in the observed fluorescence quenching. Mutations were introduced to four Trp residues in L67LAzY, and the responses were compared with that of the wt L67LAzY. Mutants L67LAzY_W41HF, L67LAzY_W52HF, L67LAzY_W55HF, L67LAzY_W118HF, and L67LAzY_W41LF were developed using site-directed mutagenesis (Figure 4a). Except for L67LAzY_W41LF, the antibodies were purified using an IgG-conjugated column. Purified antibodies were modified by DBCO-Cy3 via the 3-azido-l-tyrosine residue. The Q-bodies were resolved by SDS-PAGE and detected by fluorescence imaging of the gel (Figure 4b).

### 3.5. Antigen-dependent Changes in Fluorescence Intensity of Labeled Antibodies

**Figure 5:**
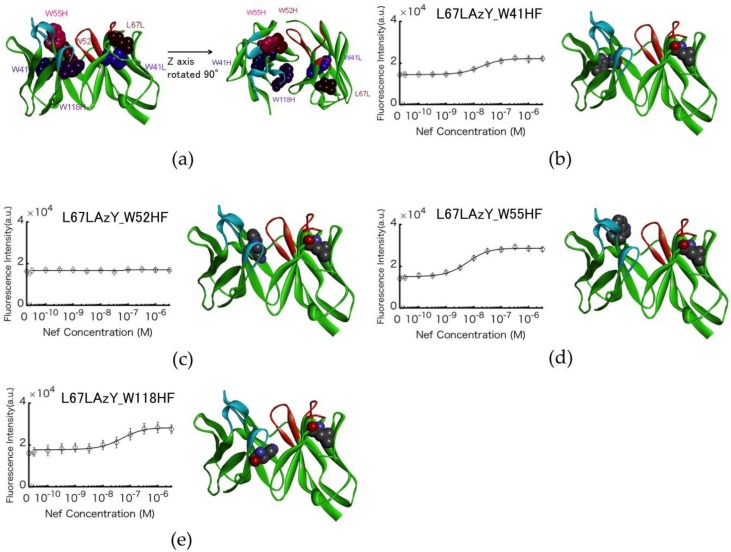
Antigen-dependent enhancement of Nef Q-body fluorescence. (**a**) Position of the mutated residues of the anti-Nef antibody. (**b**–**e**) Schematic illustration of the structure of the Nef Q-body (right). Titration curves of the normalized fluorescence intensity of the Nef Q-body (left). Error bars represent ±1 S.D. (n = 3).

Four types of labeled antibody were mixed with 0–3.2 µM Nef wt. The change in fluorescence intensity of L67LAzY_W41HF and L67LAzY_W52HF was smaller than that of L75AzY (Figure 5a–e), whereas L67LAzY_W118HF and L67LAzY exhibited a change of similar magnitude. The change in fluorescence intensity of L67LAzY_W55HF was greater than that of L67LAzY.

### 3.6. Analysis of the anti-Nef Antibody Epitope

**Figure 6:**
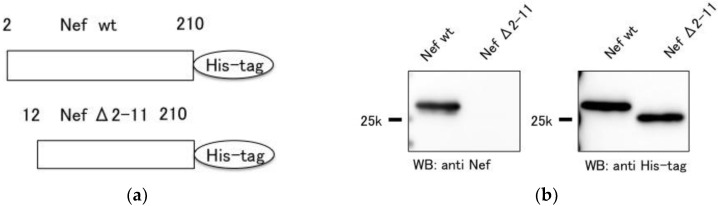
Determination of the epitope of the anti-Nef antibody. (**a**) Schematic illustration of Nef wt and NefΔ2-11. (**b**) Western blotting with the anti-Nef antibody (left) and anti-His-tag antibody (right).

To determine the epitope of the anti-Nef antibody, Nef Δ2-11 was produced by deletion of 10 residues at the N-terminus of Nef wt (Figure 6a). The anti-Nef antibody recognized Nef wt but not Nef Δ2-11, suggesting that the epitope of the anti-Nef antibody is located within residues 2–11 of the antigen (Figure 6b).

### 3.7. Decrease in Response Resulting from Mutation of the Antigen (Nef)

**Figure 7:**
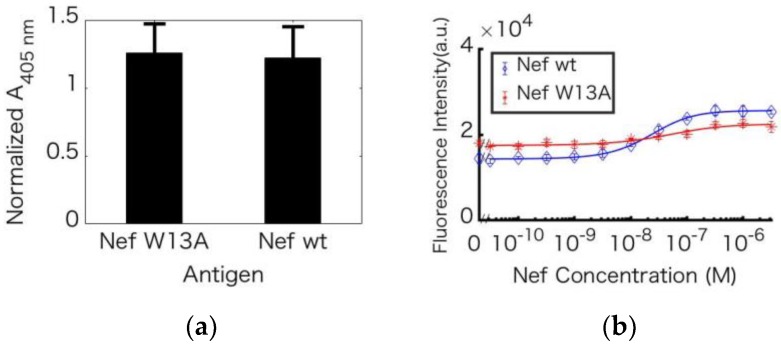
Antigen-dependent enhancement of fluorescence of the Nef Q-body with mutated Nef. (**a**) Specific binding of L67LAzY to Nef wt and Nef W13A. (**b**) Titration curve of the fluorescence intensity with Nef wt (blue line) and Nef W13A (red line). Error bars represent ±1 S.D. (n = 3).

A Trp-to-Ala mutant of the antigen, Nef, was constructed to elucidate the involvement of Trp residues in the observed fluorescence quenching. Nef wt and Nef W13A were expressed and purified using a Ni column. To confirm that the anti-Nef antibody recognized W13 of Nef, the affinity between Nef wt and Nef W13A was compared using ELISA (Figure 7a), and the results showed no significant difference in affinity. L67LAzY was mixed with 0–3.2 µM Nef wt or Nef W13A, and the change in the fluorescence intensity of L67LAzY with Nef W13A was lower than that with Nef wt (Figure 7b).

## 4. Discussion

In this study, Q-bodies were developed from anti-Nef antibody. Various amino acid residues of the antibody were changed to the UAA 3-azido-l-tyrosine with DBCO-Cy3 modification of the azido group. The fluorescence of the labeled antibody was found to be antigen dependent. L67LAzY exhibited the greatest change in fluorescence intensity (1.8-fold). However, the change in fluorescence intensity of T66LAzY was only 1.4-fold, despite the nearly identical position of the modification. Homology modeling predicted that T66L and L67L form a β-sheet, with the side chains oriented in opposite directions. This suggests that Q-body’s performance is related not only to the distance between the Trp residues and the fluorescent dye [5], but also to the direction of the side chain in which the fluorescent dye is incorporated. As the C-alpha of S69L is farther from the center of the CDR pocket (16 Å) than L67L (14 Å), the fluorescence of S69LAzY could not be stably quenched. Therefore, the change in fluorescence intensity of S69LAzY was lower than that of L67LAzY. As the C-alpha of S69L is from center of the CDR pocket (15 Å) and the direction of the side chain of S69L is opposite to the CDR pocket, the fluorescence of S69LAzY could not be stably quenched. As the C-alpha of S12L is from center of the CDR pocket (26 Å), the fluorescence of S12L was not quenched at all. The above observations informed development of the Q-body. Previous studies showed that fluorescence is quenched when the dye is near the CDR of the antibody, and the present results agree, given that the fluorescence intensity of S12LAzY did not change. Therefore, quenching of the fluorescent dye occurs only if the dye can bind the CDR. This result also suggests that the direction of the side chain is important. Therefore, consideration of multiple incorporation positions is necessary in developing higher-performance Q-bodies with optimal position and direction of the fluorescent dye.

The amino acid residues involved in quenching the fluorescence of L67LAzY were examined. According to the abYsis antibody database [25], the rates of conservation of the four Trp residues W41H, W52H, W118H, and W41L are high (100%, 94%, 99%, and 100%), but that of W55H is low (10%). The rate of change in fluorescence intensity of the two mutants L67LAzY_W41HF and L67LAzY_W52HF was lower relative to the other mutants. The fluorescence intensity of the L67LAzY_W118HF mutant with the conserved Trp residue did not change, whereas that of the W55H mutant, in which the Trp residue was not conserved, increased (2-fold). It can detect Nef over 3.2 nM. According to the predicted model, the solvent-accessible surface is low for W41H, W52H, and W118H (0%, 2.5%, and 12%, respectively), but that of W55H is relatively high (17%), and W55 is located on the same side as the CDR. These data suggest that fluorescent dye excluded from the CDR pocket by the antigen-binding bound to the unconserved Trp residue W55H and was quenched. The fluorescent dye of L67LAzY_W118HF was not quenched, as the W118H was located far from L67L and thus did not affect the fluorescence intensity. The decrease in the fluorescence intensity of mutant L67LAzY_W41HF suggests that W41H interacts directly with the dye, resulting in quenching. L67LAzY_W52HF did not exhibit change of the fluorescence intensity. Although the side chain of W41H is embedded in the VH region, the side chain of W52H is exposed in the CDR pocket. Therefore, L67LAzY_W52HF could exhibit stronger quenching of the dye than L67LAzY_W41HF. Conserved Trp residues are usually related to folding of the antibody, suggesting that mutagenesis of Trp residues affected antibody folding. Our results suggest that quenching of the fluorescent dye is related to the number of the Trp residues and their distance from the dye as well the positional relationship between the Trp residues and the dye. Based on the above, the following factors appear to have the greatest effect on Q-body performance. Trp residues near the CDR that are not involved in binding to the antigen or folding must be removed. Retention of conserved Trp residues to the greatest degree possible is necessary to ensure quenching of the fluorescent dye. Also, because concentration of the Q-body has not been optimized, more superior efficiency might be obtained after the optimization.

Western blotting with the Nef mutant showed that the anti-Nef antibody recognized the Nef 2–11 region. The results of ELISAs with Nef_W13A indicated that the anti-Nef antibody does not recognize W13, since we have shown that the antibody binds equally well to Nef_W13A and Nef_wt. As Trp residues affect quenching of fluorescence, fewer Trp residues in the antigen result in greater differences in fluorescence intensity. However, the fluorescence intensity decreased with Nef_W13A. We hypothesize that the mutagenesis caused quenching mediated by another amino acid residue. In addition to Trp, His, Met, and Tyr residues can quench fluorescent dyes [26]. However, there are no such amino acid residues near W13. Mutagenesis did not affect the local structure near W13 (PDB code 1QA4 [27]), as this region does not form secondary structure. These observations suggest that mutagenesis caused a change in the antibody-antigen complex structure and the antigen’s local structure located far from W13. Although it was thought that fewer Trp residue would result in a greater change in fluorescence intensity, the results did not support this hypothesis. However, it was confirmed that antigen mutagenesis outside the epitope region affects fluorescence intensity.

Q-bodies as reagents that are capable generating a signal directly upon target binding in a single step manner are supposed to work with some electrochemical-based sensor platforms. For example, in the case of some aptamer-based sensors, there is a great need for additional affinity reagents, and Q-bodies could work with such approaches. By the versatility like this, applications of Q-bodies are expected to be extended in the future.

## 5. Conclusions

In this study, a Nef Q-body exhibiting a 2-fold antigen-dependent fluorescence intensity change was developed using an in vivo UAA incorporation system. The sensitivity of the Q-body was related to the number of Trp residues as well as the direction of the amino acid residues incorporated in relation to the fluorescent dye. A Trp residue at the surface of the antibody could quench the fluorescent dye via direct interaction. Furthermore, antigen residues also affect fluorescence intensity. These results should facilitate future development of a Q-body exhibiting higher sensitivity than those currently available.

## Figures and Tables

**Table 1:** Nucleotide sequences of primers used in the present study.

Primer Name	Nucleotide Sequence (5′-3′)
303_T66LAzY_F	ctacgccgtttcctagttagattctggtgt
303_T66LAzY_R	acaccagaatctaactaggaaacggcgtag
303_L67LAzY_F	cgccgtttccacttaggattctggtgtccc
303_L67LAzY_R	gggacaccagaatcctaagtggaaacggcg
303_S69LAzY_F	ttccactttagattagggtgtcccaaaaag
303_S69LAzY_R	ctttttgggacaccctaatctaaagtggaa
303_D101LAzY_F	tgaagattttgcatagtattactgtctcca
303_D101LAzY_R	tggagacagtaatactatgcaaaatcttca
303_S18LAzY_F	tccatcctccttataggcctctctgggaga
303_S18LAzY_R	tctcccagagaggcctataaggaggatgga
303_W41HF_F	cgactatatgcacttcgtgaagcagaggcc
303_W41HF_R	ggcctctgcttcacgaagtgcatatagtcg
303_W52HF_F	acagggcctggagttcattggatggattga
303_W52HF_R	tcaatccatccaatgaactccaggccctgt
303_W55HF_F	ggagtggattggattcattgatcctgagaa
303_W55HF_R	ttctcaggatcaatgaatccaatccactcc
303_W118H4F_F	tacaagggatgtcttcggcgcagggaccac
303_W118HF_R	gtggtccctgcgccgaagacatcccttgta
303_W41LF_F	tggttacttaagcttccttcagcagaaacc
303_W41LF_R	ggtttctgctgaaggaagcttaagtaacca
Nef_del2to11_F	agtagtccatgggatggcctgctgtaagg
Nef_R	aagtgtagcggtcacgctgcgcgtaaccac
Nef_W13A_F	tagtgtgattggagcacctgctgtaaggga
Nef_W13A_R	tcccttacagcaggtgctccaatcacacta

**Table 2:** Template and primers used for the construction of the gene.

Gene	Template	Primers
T66LAzY	wt anti-Nef antibody	303_T66LAzY_F, 303_T66LAzY_R
L67LAzY	wt anti-Nef antibody	303_L67LAzY_F, 303_L67LAzY_R
S69LAzY	wt anti-Nef antibody	303_S69LAzY_F, 303_S69LAzY_R
D101LAzY	wt anti-Nef antibody	303_D101LAzY_F, 303_D101LAzY_R
S18LAzY	wt anti-Nef antibody	303_S18LAzY_F, 303_S18LAzY_R
L67LAzY_W41HF	L67LAzY	303_W41HF_F, 303_W41HF_R
L67LAzY_W52HF	L67LAzY	303_W52HF_F, 303_W52HF_R
L67LAzY_W55HF	L67LAzY	303_W55HF_F, 303_W55HF_R
L67LAzY_W118HF	L67LAzY	303_W118HF_F, 303_W118H4F_R
L67LAzY_W41LF	L67LAzY	303_W41LF_F, 303_W41LF_R
NefΔ2–10	Nef wt	Nef_del2to11_F, Nef_R
Nef W13A	Nef wt	Nef_W13A_F, Nef_W13A_R
